# Barriers to Advance Care Planning at the End of Life: An Explanatory Systematic Review of Implementation Studies

**DOI:** 10.1371/journal.pone.0116629

**Published:** 2015-02-13

**Authors:** Susi Lund, Alison Richardson, Carl May

**Affiliations:** 1 Faculty of Health Sciences, University of Southampton, Southampton, United Kingdom; 2 Royal Berkshire NHS Foundation Trust, Reading, United Kingdom; 3 University Hospital Southampton NHS Foundation Trust, Southampton, United Kingdom; 4 National Institute for Health Research Collaboration for Leadership in Applied Research and Care Wessex, Southampton, United Kingdom; University of Glasgow, UNITED KINGDOM

## Abstract

**Context:**

Advance Care Plans (ACPs) enable patients to discuss and negotiate their preferences for the future including treatment options at the end of life. Their implementation poses significant challenges.

**Objective:**

To investigate barriers and facilitators to the implementation of ACPs, focusing on their workability and integration in clinical practice.

**Design:**

An explanatory systematic review of qualitative implementation studies.

**Data sources:**

Empirical studies that reported interventions designed to support ACP in healthcare. Web of Knowledge, Ovid MEDLINE, CINAHL, PsycINFO, British Nursing Index and PubMed databases were searched.

**Methods:**

Direct content analysis, using Normalization Process Theory, to identify and characterise relevant components of implementation processes.

**Results:**

13 papers identified from 166 abstracts were included in the review. Key factors facilitating implementation were: specially prepared staff utilizing a structured approach to interactions around ACPs. Barriers to implementation were competing demands of other work, the emotional and interactional nature of patient-professional interactions around ACPs, problems in sharing decisions and preferences within and between healthcare organizations.

**Conclusions:**

This review demonstrates that doing more of the things that facilitate delivery of ACPs will not reduce the effects of those things that undermine them. Structured tools are only likely to be partially effective and the creation of a specialist cadre of ACP facilitators is unlikely to be a sustainable solution. The findings underscore both the challenge and need to find ways to routinely incorporate ACPs in clinical settings where multiple and competing demands impact on practice. Interventions most likely to meet with success are those that make elements of Advance Care Planning workable within complex and time pressured clinical workflows.

## Introduction

People with end-stage disease may find their capacity to communicate their wishes about whether or not to continue with life sustaining treatments, or undergo resuscitation in the event of a cardiopulmonary arrest, threatened by pathophysiological problems. In such situations patients and their families may experience heroic but sometimes distressing efforts to ensure survival, or continued treatment programmes in which physical comfort and personal dignity become difficult to secure [1]. Advance Care Plans (ACPs) [2,3] are one means to ensure that patient and family preferences are negotiated, identified and recorded before the patient is overtaken by disease. They offer a means through which patients can make clear—ahead of time—their preferences for the future including treatment in the final days, hours and moments of life. They have important benefits, enabling clinicians and caregivers to resolve uncertainties about what patients would or would not want, and to specify plans for action or inaction [4].

Although there is a substantial body of research, clinical practice, and patient experience that seems to support their use, ACPs are frequently framed as solutions for an ethical problem (to do with patient autonomy), or for relational difficulties (to do with the complex network of negotiations and interactions that take place in the face of impending loss of self) [5]. In this paper, we take a different tack. We consider the operationalization of ACPs as an implementation problem, and focus on a single research question: what factors promote or inhibit the routine incorporation of ACPs in clinical practice? To answer this question, we undertook a literature review that focused attention on the dynamics of implementation and investigated the effects of known mechanisms of workability and integration[6–8].

This is the first systematic review of ACPs to focus on implementation problems. It is also the first to be informed by implementation theory. Normalization Process Theory (NPT) [9,10] is a formal grounded theory that characterizes a set of mechanisms that have been empirically demonstrated to be important to the behaviour of clinicians [11,12] and patients [13,14]. It provides a useful framework for explanatory systematic reviews [6–8,12]. NPT proposes that four key mechanisms (coherence, cognitive participation, collective action, and reflexive monitoring) are associated with the investments that people make in meaning, commitment, enacting, and appraising new or modified ways of conceptualizing, enacting, and organizing practice. NPT focuses on action—the things that people *do* rather than their attitudes or beliefs about practices—and explains how the operation of these key mechanisms promotes or inhibits the routine embedding of innovations in everyday work.

## Defining Advance Care Plans

The fundamental purpose of an ACP is to represent the wishes of the patient in the face of future circumstances that may mean that they are denied the opportunity to state those wishes. In such circumstances, which tend to be legally defined [2,15] around loss of mental capacity to make and communicate important decisions, an ACP may perform one or more of the following explicit functions:

- Provide an opportunity to consider existential and relational aspects of impending loss of self at the end of life.
- Provide an opportunity to clearly acknowledge the prospect of death, and in that context to negotiate personal preferences about future treatment decisions between patient, family members and clinicians.
- Make clear a patient’s preferences about clinical actions that will follow their loss of capacity (for example, about continuation of treatment or resuscitation).
- Delegate responsibility for implementing a patient’s preferences to those agents of a healthcare system who have legal responsibility (and liability) for the conduct and delivery of care; agents of other social institutions (for example, lawyers or priests); and to family members or other caregivers.
- Make clear the patient’s preferences for the administration of their person and property during a period of loss of capacity, and during and after death, and to negotiate these with health professionals, family members, and other caregivers.

It is immediately clear that ACPs bring in their wake emotionally, clinically and legally complex problems. Importantly, they may not always be binding in law, and even if they are, they may be contested, or overruled, by family members and health care professionals [16,17]. Loss of capacity is the key to the clinical and legal constitution of ACPs and in many countries, legislation has empowered patients to refuse treatment when they come to regard it as futile or intolerable [16,18]. In this context what Glaser and Strauss [19] called *awareness contexts*, (the extent to which patients are deemed to know about their impending death, and the significance of negotiating this awareness), have become important underpinnings of patients’ autonomy. It must be clear to the patient—and to their significant others—that such a decision is necessary, and that mechanisms exist to make this possible. Such decisions are regularly made by cognitively intact patients in the face of specific treatment decisions—for example, about stopping chemotherapy for cancer, withdrawing from dialysis for renal disease, or switching off implanted electronic devices in heart failure [20]. The assumption that underpins the ACP is quite different: it is that there may be a point at which the patient is not a cognitively intact participant in a decision-making process about their care at the end of life.

The negotiation of an ACP between a patient, family, and clinicians is therefore a good deal more than a personal, existential, set of decisions. It is both a *rite of passage* that defines a person’s shifting identity and clinical status in relation to both the self and others, and a *procedural device* intended to reduce uncertainty about the actions that different groups will take in response to that status in a specific set of clinically defined circumstances [15]. All of this is set in a complex medico-legal structure that may be defined by specific legislation [18]. Against this background, discussion of ACPs tends to be framed in two ways.

The first approach seems to be psychosocially oriented: it suggests that ACPs should be patient driven; are best undertaken when the patient is in their own environment; should be started early rather than in a time of crisis; and should be focused on broad goals and preferences that may or may not be documented [21]. This is a kind of phenomenological approach that is aimed at thinking about the future.

The second approach is more pragmatic. Following a trigger event such as diagnosis, recurrence, or hospital or care home admission it recognises that a significant transition has occurred [22,23]. It then focuses on identifying the patient’s specific goals and preferences about care in the face of death. These are then documented and shared with family and health professionals. This is a more organisationally oriented approach that is intended to form a starting point for action.

We can hypothesise that patterns of negotiation for ACPs, and their orientation, are related to each other. They may depend on the dimensions of what is to be negotiated with the patient, and the functional orientation of the ACP itself. In turn, this raises an important question. What is actually to be implemented? We take the simplest definition that we can.

- An ACP is an interactional process between a patient, significant others, and clinicians. Within this interactional process cognitive and communicative incapacity, and loss of self at end of life, are acknowledged as a future risk. In response to this risk, the patient’s preferences about clinical and other actions are expressed, negotiated, acknowledged and recorded by other participants in the expectation that they will be acted upon if necessary.

In this definition, an ACP is an interactional process that decides upon possible future actions, and mechanisms to ensure that these decisions are available to those who might be asked to take those actions. The means of translation of preferences and decisions into action are consequences of ACPs rather than components of them. It is important to emphasise that when we speak of implementing ACPs we are not concerned with the operationalization of the ACP in the cases of individual patients, but instead focus attention on what is known about their embedding in healthcare practice.

## Methods of Review

**Fig 1:**
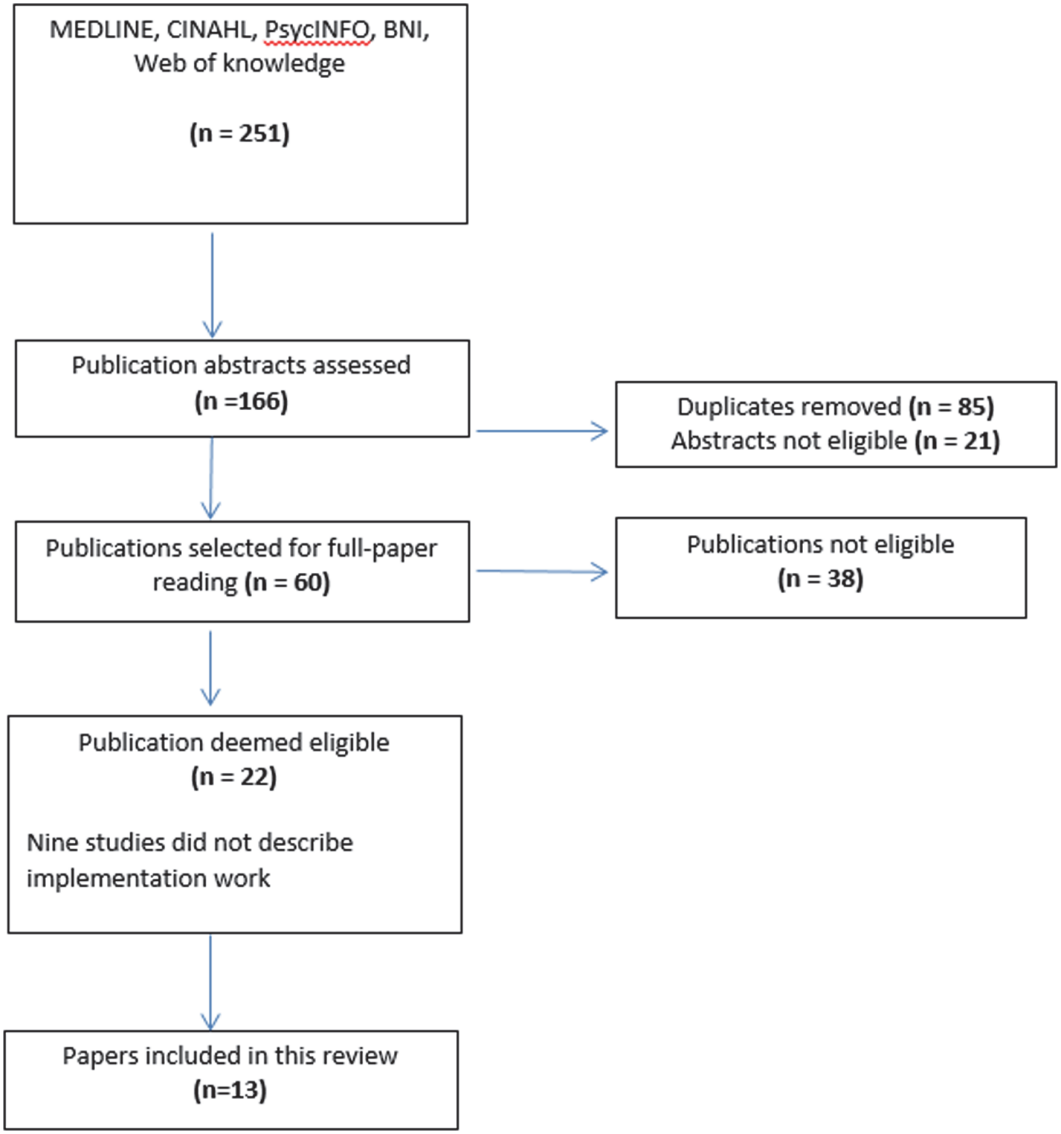
PRISMA flowchart.

We undertook an explanatory review of literature describing the operationalization of ACPs (see Fig. 1.). The principal inclusion criterion for the review was that papers should report the implementation of interventions intended to support Advance Care Planning in healthcare settings. We excluded interventions aimed at children and those with mental health problems because these groups are not framed as legally competent in most legal systems. We did not exclude reports on the grounds of methods (our review includes reports of implementation studies that used different methods), and we did not exclude studies on grounds of methodological quality. Uneven methodological quality and poor reporting of intervention studies are commonly found in reviews, but in the absence of agreed standards for reporting implementation interventions exclusing such papers is likely to be arbitrary and may skew the literature still further [24]. We searched Web of Knowledge, Ovid MEDLINE, CINAHL, PsycINFO, British Nursing Index and PubMed databases. Broad truncated terms for searching study abstracts were piloted until a combination of terms began to identify studies relevant to this review (advance care plan* and end of life or palliative, and implement*). The search was not restricted to specific years in order to capture the evolution of the ACP literature over time. Empirical studies in English and related to adults were included. References of studies selected for full examination were scanned for further relevant studies. Manual searches of relevant journals were also undertaken, along with a review of advance care planning websites. Studies were eligible for inclusion if they presented primary research or findings from clinical implementation or evaluation.

Papers selected for the review are described in Table 1. They were treated as qualitative data. We undertook directed content analysis [25] of the results and discussion sections of each paper, using an analytic framework based on that used in an earlier review [6] informed by Normalization Process Theory. A data extraction proforma was developed using NPT to structure data analysis (see Table 2) In treating papers as qualitative data, we focused only on direct claims about implementation processes relating to advance care plans, the factors that promote and inhibit these, and the contexts in which they took place. Included studies were searched (by SL and CRM) for evidence that provided a description of elements of the implementation process. For evaluation studies and those focused on professionals this was often scarce or missing, however, these were also included if significant barriers or facilitators to implementation were illuminated.

**Table 1:** Studies included.

Publication details	Principal objective	Context	Participants	Study design
Baker et al (2012) (31) UK	To identify a population at risk of admission to hospital and to provide an anticipatory care plan.	General practice and primary care team, Scotland. Patients identified using Nairn case finder.ACP introduced through proactive case management.	Patients. 96 in ACP arm, 96 matched cohort	Prospective cohort study of service intervention. Outcomes: Admissions,Occupied bed days, costs
Boyd et al (2010) (28) UK	To assess feasibility of implementing ACP in UK primary care	Primary care. Educational workshop about ACP directed at healthcare professionals, a toolkit of resources including ACP document.	4 GP practices in Scotland. 20 GP’s, 8 community nurses, 9 telephone interviews with GP’s with special interest in palliative care	Retrospective. Mixed method evaluation of pilot intervention. Outcomes: Effectiveness of workshop. Change in clinician attitude to ACP
Bravo et al (2012) (29) Canada	To assess efficacy of a multi modal professionally led ACP intervention	Community based dyads of older adult and their self selected proxy. Trained facilitator using clinical vignettes to elicit preferences and assess ability of proxy to predict preferences. Opportunity to complete ‘My Preferences’ document	Patients and their proxy. 240 dyads in 2 groups—experimental or control	Prospective. Single blind stratified randomised control trial study protocol. Prediction accuracy measured at baseline, at end of intervention and 6 months post intervention. Outcomes: Concordance assessments. Cost analysis
Detering et al (2010) (32) Australia	To investigate impact of ACP on end of life care in elderly patients	Acute Hospital Inpatients aged 80 plus. ACP actioned by trained facilitator using Respecting Choices tool.	309 patients usual care or usual care plus ACP. 154 in intervention group.	Prospective RCT. Interviews and case note review. Outcomes: Whether a patient’s end of life wishes were known and respected. Patient and family satisfaction. Stress, anxiety and depression in relatives
Hockley et al (2010) (33) UK	To investigate impact of implementing 2 end of life care tools	Nursing homes in Scotland. Facilitator plus 2 champions for each home supported implementation of Gold Standards framework and Liverpool care pathway	7 private nursing homes. 228 residents notes examined. Staff questionnaire 68	In depth evaluation utilising case note review of patient outcome, staff perceptions of implementation and interviews with bereaved relatives.
Jeong et al (2007) (35) and (2010) (34) Australia	To investigate phenomenon of ACP and use of advance care directives in residential aged care facilities	Residential aged care facilities. ACP facilitated by clinical nurse consultant.	3 residential care facilities. Interviews with 3 residents, 11 family members and 13 nursing staff	Prospective case study using participant observation, field notes, semi structured interviews and document analysis. Outcome; Staff knowledge of ACP. Identification of components and factors involved in ACP. Development of a conceptual framework for ACP implementation
Matlock et al (2011) (30) USA	To assess feasibility and acceptability of decision aid designed for patients facing advanced or terminal illness	University of Colorado hospital, inpatient palliative care unit. Standard palliative care consultation plus copy of decision aid.	51 patients or decision makers	Prospective pilot randomized clinical trial of a decision aid. Outcomes: Decision conflict and knowledge
Sampson et al (2011) (39) UK	To design and pilot a palliative care and ACP intervention	Patients who had an acute hospital emergency admission and dementia. Palliative care assessment informed an ACP discussion with the carer.	33 patients and carers. 22 intervention and 11 control	Explanatory RCT. Pilot study involving a palliative care assessment. Outcomes: Carer related—distress levels. Decisional conflict satisfaction levels. Patient—pain and distress (carer rated)
Schiff et al (2009) (38) UK	To design and evaluate a document (Expression of Healthcare Preferences (EHP)) to enable older inpatients to discuss and record end of life healthcare preferences	Acute NHS hospital. Design of a document and evaluation of its use and acceptability	95 patients given EHP. 56 feedback questionnaires	Prospective questionnaire and outcome evaluation. Outcomes: Number and content of completed EHPs. Patient feedback
Seymour et al (2010) (27) UK	To identify factors surrounding community nurses’ implementation of ACP and nurses’ educational needs	Community nurses from 2 cancer networks, focus group discussions about end of life care and their views about ACP	23 community nurses	Qualitative using action research. Focus groups and workshops.Outcome: Improved understanding of community nurses understanding and implementation of ACP.
Shanley et al (2009) (36) Australia	To gain an understanding of how ACP is understood and approached by managers of residential aged care facilities	Managers from 41 residential aged care facilities from South Western Sydney. Discussion of current approach to ACP	41 managers	Qualitative using semi structured interviews Outcome: Increased understanding and development of a continuum model of practice
Simon et al (2008) (40) Canada	To gain an understanding of patient experience of facilitated ACP	Facilitated ACP with outpatients with end stage renal disease using the Respceting Choices tool	Six patients	Prospective study using grounded theory. Interviews. Outcome: Identification of 3 major categories to explain how participants experienced facilitated ACP

**Table 2:** Coding frame and Taxonomy Items.

**Coding Frame**			**Taxonomy Items**
NPT Construct	Process	Observable action	
Coherence	Participants attribute meaning to the activities that surround a practice and make sense of its possibilities within their field of agency.	What do clinicians do to make sense of the ACP and work out how to put it into action?	Push to reduce unwanted hospital admissions at end of life [30,31,35]; operationalize proactive models of care [27]; act to increase or preserve patient autonomy and minimize proxy decision-making [28,31,33–35,38]; act to reduce stress and anxiety amongst patients and family members [27,28]; improve end of life care in residential facilities and nursing homes [32–35,38]; act to operationalize policy within a clinical setting [26,37]; resolve confusion over policy and legislation [39].
Cognitive Participation	Participants legitimize and enroll themselves and others into activities around a practice.	What do clinicians do to become ACP users, and to commit—or otherwise—to its use?	Mobilize support for reactive case management [30]; employ a specialist facilitator to deliver the intervention[27,28,31–34]; build a team around ACPs [30]; educational workshops to build support for ACP [27]; availability and turnover of staff [27,31,35,36]; nomination of significant others empowered to participate in decision-making [31,38].
Collective Action	Participants mobilize skills and resources needed to enact a practice.	What do clinicians do to use the ACP in practice, and what do they do to become skilled and resourced practitioners?	Robust mechanisms for case-finding and identification of unmet need [27,30,31]; Developing communications skills and opening up interactional opportunities [26,27,31,36,38,39]; utilizing shared resources for professionals, and patients [26,27,32,35,37,39]; integration of information within and between organizations [26,27,31,32,35]; integrating paper based systems with IT systems [27]; increasing frequency of written plans [28,31].
Reflexive Monitoring	Participants assemble and appraise information about the effects of a practice and utilize that knowledge to reconfigure relationships and behaviors.	What do clinicians do to evaluate and the effects of the ACP, and how to they translate the results of this into practice?	Prognostic uncertainty [27,28,39]; concordance/adherence assessments [28,31]; assessment of impact of ACP on care offered to patients and families [31–34,39]; demand for greater organizational responsiveness [26,30]; concern about need to support patients and families through prognostic uncertainty and anxiety [27,38,39]; opposition to increased bureaucratization [27]; need to improve timeliness of ACP intervention [26,35,38].

Directed content analysis involved the following procedures. We operationalized key constructs of Normalization Process Theory as an analytic framework, modifying a framework developed for an earlier systematic review [6]. Second we translated the framework into a coding frame, and searched the text of included papers’ results and discussion sections for information about factors that promoted or inhibited implementation of ACPs. We then created a taxonomy of such factors, relating them to intervention types, clinical contexts, and healthcare systems. Members of this taxonomy were then clustered into related groups, and the phenomena that they described were then characterized in a set of propositions and mapped onto a set of conceptual models of ACP implementation. The coding frame and taxonomy items derived from it are described in Table 2.

## Results: Factors that Affect the Implementation of Advance Care Plans

We identified 251 citations. After duplications were removed and abstracts reviewed for appropriateness, 166 abstracts were screened and 60 papers were then selected for full review. Two authors (SL and CRM) then read and checked all papers, eliminating those that did not describe the implementation process. Thirteen papers were finally determined to meet the principal inclusion criterion, and these described twelve studies. These dated from 2007 to 2012. Six were from the UK, two from Canada, one from the USA and four from Australia. Qualitative methods were used in all studies with half focusing on health care professional and half on patient and family.

Studies included in this review all took as their point of departure the proposition that ACPs should be initiated by health professionals to solve a problem for patients and their relatives [26]. Indeed, the professional orientation of this work was a taken-for-granted element of all of the papers reviewed. In this context the work of delivering ACPs was contextualized in two main ways. Implementing ACPs could answer a problem of urgent demand, because of the need to respond urgently to diagnosis or prognosis [27–29], or to avoid readmission to hospital [30]. But implementing ACPs could do more than answer an urgent problem. It could also create a framework that routinizes practice by operationalizing a consistent approach [31–35], and by utilizing an electronic health record or other administrative system [36,37].

All studies in this review sought to evaluate a response to these operational problems for health service providers and it is not surprising that responses—however they were evaluated—were highly structured. Negotiating ACPs is an interactionally risky business, and to mitigate this risk studies reviewed employed a structured discussion, educational intervention, or toolkit. These included risk stratification tools [30]; structured preference-elicitation and decision-making tools [27–29,31–34,37–39]; treatment escalation plans [36]; conversational frameworks and shared documentation in the community [26,35]. In consequence, interventions tended to be delivered by specially prepared professionals with a clear role as facilitator. This was especially the case in those studies where highly structured preference-elicitation and decision-making tools were utilized. Others involved attempts to incorporate a wider team [35], or even resisted attempts to standardize ACPs in practice [27].

The interventions evaluated in these studies had three main features. They had an operational focus; and consisted of operationalizing a standardized and structured intervention; and employed specially prepared facilitators. A key factor that promoted the implementation of these interventions was that they were evaluation studies supported by research infrastructure. The availability of this infrastructure meant that in some studies additional resources were available to staff to support the advance care planning process. Most of the papers reviewed here [27–37,39], reported on prescheduled interventions and administrative procedures applied to selected patients; dedicated teams to champion and organize interventions, and specially trained facilitators to deliver them. Recalling our earlier distinction between components of ACPs that form an interactional process, and components that form structural mechanisms to support the process, we can see that factors that promoted implementation in these studies were largely those concerned with structural mechanisms such as a dedicated document and organisational policies and guidelines to support the process.

**Fig 2:**
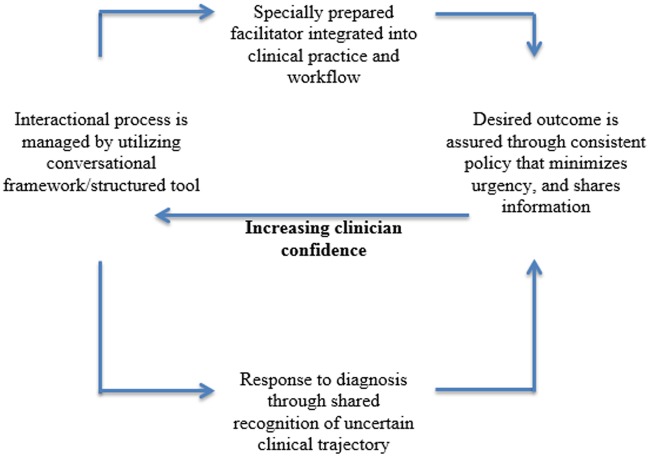
Operationalizing Advance Care Plans: Facilitators.

**Fig 3:**
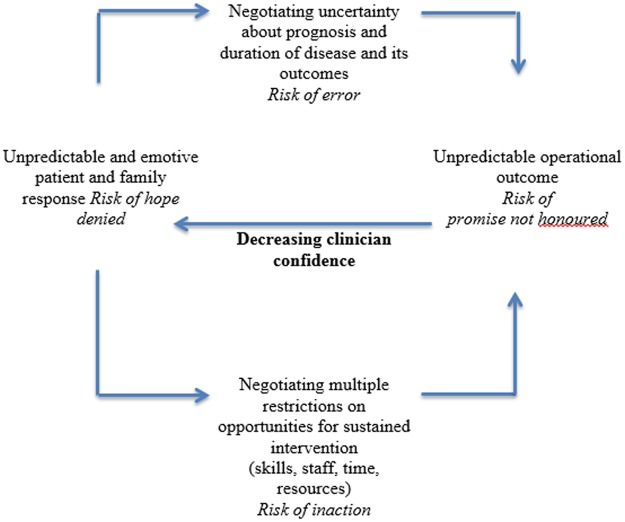
Operationalizing Advance Care Plans: Barriers.

In Figs. 2 and 3 we have characterised the factors that promote or inhibit the implementation of ACPs. We mapped them as elements of a clinical process, in which the outcomes are not those that matter to individual patients but are those that ensure increasing or decreasing confidence in the mechanisms through which ACPs are delivered in practice. The focus here is on the management of problems of uncertainty in professional-patient interactions by implementing structured tools and plans of action that provide a script for professionals and organizations to follow. These scripts appear to share some of the characteristics and problems previously observed in clinical guidelines and shared decision-making tools [40]. While uncertainty is important, however, it is not necessarily the principal barrier to effectively implementing ACPs. The main problem of implementation is likely to be from competition with other important—and sometimes intensively monitored—clinical and organizational tasks. In this context, complex and time consuming interactions with patients, especially those with uncertain and emotive outcomes, can quite quickly become incompatible with the other demands on clinicians’ time. A cadre of specialist facilitators is easily seen as the answer to this, but may place new demands and costs on the processes of clinical care, even though utilizing specialists is the most effective way of *researching* and *evaluating* ACPs. After all, barriers and facilitators are not opposites of each other. This means that doing more of the things that facilitate the delivery of ACPs may not reduce the effects of those things that undermine them.

Examining the factors that inhibited implementation of these interventions reveals the important part likely to be played by unstarted, incomplete, or failed interaction processes. Problems associated with these begin with prognostic uncertainty and its effects. The illness trajectory of a specific patient cannot be estimated accurately [27,28,39], and this also creates a problem of timing the delivery of ACPs [29]. This adds difficulty to the already emotionally and practically complex business of interactions between patients (and their families) and clinicians that raise questions about forthcoming death [26,36,38], and which may undermine patients’ positive coping strategies, which may include hope for the future, even when the latter is unrealistic [27,38]. The workability of ACP interventions was seen to rest on improving communication skills [27,33,34,36]. But even if communication skills are improved, these studies pointed to barriers to integrating ACPs in clinical workflows. These included the time consuming nature of interventions [31]; the limited numbers, and turnover, of trained staff available to initiate, perform and implement them [27,31,35,36]; and problems in sharing decisions, preferences and plans across health economies and embedding them in patients’ health records in an accessible way [26,27,31,32,35].

## Discussion

Systematic reviews are typically oriented to reporting the results of summative studies. Ours has focused on formative information about implementation processes. However, it has important limitations. Across the body of literature that might have contributed to this review, much useful information about the processes of implementing and delivering ACPs is missing. (See, for example, Fagerlin and Schneider for a review of the failure of the living will [41].) The literature in this field is likely to be biased towards the presentation of positive results, and inadequate reporting of intervention design and methods of evaluation is common. These factors, *inter alia*, may have meant that important studies were not discovered by our literature searches. Those studies that have been included have then been interpreted using a theoretical framework different from those utilized by their authors. These are normal problems in systematic reviewing, but we are aware that they are important limitations on our analysis, and that they demand caution in inference from the data and their analytic interpretation.

We need to better understand the dynamics of implementation processes and the factors that promote or inhibit outcomes. In this case, our analysis focuses on the factors that affect the implementation and embedding of ACPs—their *normalization* [10]—in routine clinical practice. These are characterized by the following propositions.

1. *Operational contexts are under pressure*. *Clinical and organizational pressures and the availability and preparation of staff affect opportunities to initiate and operationalize complex interventions like ACPs*.

Studies in this review responded to these operational problems by using a specially prepared facilitator to ensure ACPs could be delivered and integrated into a particular context without making demands on the clinical practice and workflow of others. This smooths the path of research, but may not be a practical proposition once an evaluation comes to an end. Where clinicians have discretion about the content of their interactions with patients and relatives they may choose the least disruptive path.

2. *Patient trajectories are uncertain. Prognostic uncertainty is an important factor that affects the clinical decision to initiate discussion of ACPs with patients and their significant others.*

Studies in this review responded to uncertainty by utilizing interventions that emphasized shared recognition of uncertainty about the duration of disease or the other events that might lead to operationalizing the ACP. This presents clinicians with the problem of differentiating between patients whose future trajectory seems clear (for example in end stage cancers, renal disease or liver failure) and those—like Chronic Heart Failure and Chronic Obstructive Pulmonary Disease—where the outcome of exacerbation events is unpredictable [42]. The latter may be less likely to be offered the opportunity to consider an ACP.

3. *Negotiations have unpredictable outcomes. Responses of patients and their significant others to the initiation of ACPs are unpredictable and emotionally complex.*

This means that interactions between patients and staff can be demanding and potentially stressful. Studies in this review responded to unpredictability by utilizing scripts—conversational frameworks, preference elicitation and decision-making tools—that directed interactions towards specific goals. Again, this presents clinicians with a problem of differentiation, between those patients and relatives who they believe are likely to be accepting of an ACP and those who they believe are likely to be resistant to them.

4. *Advance Care Plans may not be actioned. The operational outcome of ACPs are unpredictable because clinical and organizational factors that intervene to confound elicited plans and preferences.*

Studies in this review responded to these problems by emphasizing the importance of timely preference-elicitation and decision-making, ensuring the results of this work are properly recorded and shared, and contextualizing these efforts in policies that are consistently enacted and applied. Of course, significant others may object to them, and in some cases plans and preferences may simply not be made known in time, or patients may change their mind. But where a patient is comfortable with preferences expressed through their ACP this information needs to be readily accessible and clinicians need to be willing to respond to it. Again, this is a matter where they—not the patient—have final discretion.

Except in very specific circumstances, people are reluctant to think about the circumstances of their own death, or that of those that they care for. This reluctance is usually expressed through very strong behavioural norms—embodied in the rules and conventions of everyday life and the belief systems that underpin them. Few people find it easy to contravene these rules and an important part of the social function of ACPs is to make this possible by providing an agreed and shared structure for action at a critical and complex point in the patient journey. Such decision-making processes provide a way of managing the threshold between restorative work aimed at disease, and work aimed at mitigating distress and discomfort at the end of life. In this context, ACPs perform two distinct functions. They initiate a *psychosocial mechanism—*what Glaser and Strauss called a shift in awareness contexts [19]—in which the patient’s status shifts to one characterized by the planned cessation of active treatment and death as its consequence. They also initiate a *procedural mechanism* by which those plans can be made, legitimized, recorded and shared. The outcome of psychosocial and procedural mechanisms are uncertain, and we have suggested that structured tools to reduce uncertainty are likely only to be partially effective if they are only mobilized by a small cadre of dedicated specialists. The creation of such a specialist cadre is unlikely to be a workable solution for healthcare provider organizations, especially in clinical environments where expert clinicians are at a premium. The challenge, then, is to find ways to routinely incorporate ACPs in clinical settings where staff are already under pressure, and where the relational work and continuity of care that is the foundation for complex and unpredictable conversations often takes second place to the multiple and competing demands of other work.

## Conclusion

This is the first systematic review of studies of ACPs to focus on their implementation and to be informed by implementation theory. The findings of this review suggest that the interventions the most likely to facilitate ACPs are those that will equip front line professionals to manage both the interactional processes and procedural activities involved, and will provide them with a structured framework for action. The nature of this process is critical to managing the risks of error, promises not honoured and hope denied. The negative impact of systemic failures in these processes have been revealed in detail by the review of the implementation of the Liverpool Care Pathway for dying patients in the UK which reported the requirement for sensitive communication and the need to communicate uncertainty [43,44].

The *workability* of ACPs is likely to be increased if the conversations that underpin them can be focused on a simplified decision-making tool. The Option Grids developed by Elwyn et al [11,45], to support shared decision-making processes in other—equally demanding—clinical contexts offer a useful example of the kind of tool that could be developed to support these conversations. Providing clinicians with simple tools that do not require high levels of specialist preparation is likely to increase the likelihood of their adoption and normalization in practice, and it is likely to increase patients’ willingness to engage with them. Against this background, the *integration* of APCs in clinical settings cannot be left to the discretion of teams or of individual clinicians. To achieve this, healthcare provider organizations need to find ways to make clear their commitment to identifying, recording, sharing and acting upon patient preferences and to explicitly embed these commitments in their own clinical governance procedures.

## Supporting Information

S1 PRISMA Checklist (DOC)
